# Flipping Water Orientation at the Surface of Water-in-Salt and Salt-in-Water Solutions

**DOI:** 10.1021/acs.jpclett.4c01834

**Published:** 2024-10-03

**Authors:** Chun-Chieh Yu, Kuo-Yang Chiang, Ali Dhinojwala, Mischa Bonn, Johannes Hunger, Yuki Nagata

**Affiliations:** † Max Planck Institute for Polymer Research, Ackermannweg 10, 55128 Mainz, Germany; ‡ Department of Polymer Science, The University of Akron, Akron, Ohio 44325-3909, United States

## Abstract

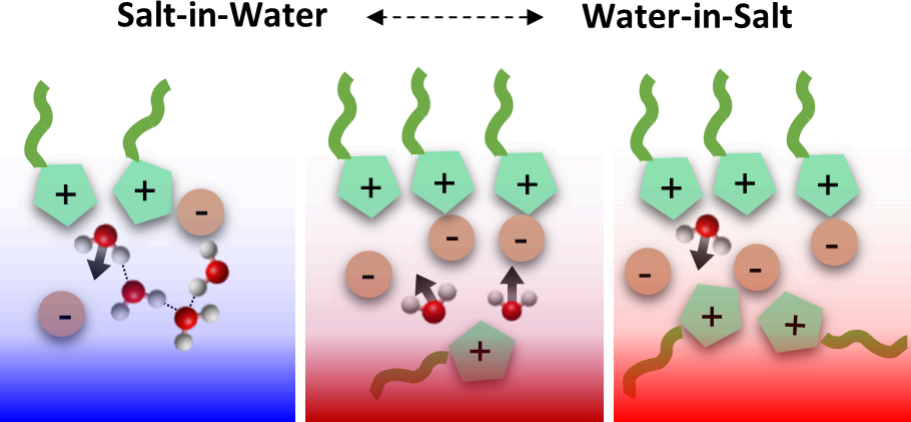

Salt-in-water and water-in-salt mixtures are promising for battery applications and fine-tuning of room-temperature ionic liquid (RTIL) properties. Although critical processes take place at interfaces of these systems, including charge transfer and heterogeneous catalytic reactions, the microscopic interfacial structures remain unclear. Here, we apply heterodyne-detected sum-frequency generation spectroscopy to aqueous solutions of imidazolium-based RTILs to unveil the microscopic structure of the interfaces of these solutions with air. Our results show that, under salt-in-water conditions, the orientation of the OH group hydrogen-bonded to the other water molecules flips from the OH group pointing down into the liquid for pure water to up due to the accumulation of anions in the cation-rich interfacial region. However, under the water-in-salt condition, the interfacial water molecules are confined by RTIL, and their orientation is down. Details of the water organization depend critically on the alkyl chain length of the imidazolium cation. Our results demonstrate that the surface structure can be tuned by altering the molecular structure and concentration of the RTIL.

Room-temperature ionic liquids (RTILs), i.e., salts that are liquid at room temperature, have been used in many research fields, including fuel cells,^1^ synthesis,^2,3^ gas separation,^4^ and electrochemical devices.^5^ Mixtures of water and RTIL have recently come into focus, because the mixtures allow for customization of the physical and chemical properties of RTILs.^6,7^ Aqueous mixtures with RTILs can be present as both, salt-in-water and water-in-salt systems, and are promising solvents for battery applications.^8−10^

To understand the electrochemical behavior of these mixtures, molecular-level insight into the structure and dynamics of the RTIL/water mixture has been gained in the bulk.^11−16^ In contrast, the molecular behavior at interfaces is challenging to address experimentally.^17^ Insights into the interfacial structure and dynamics are important, given that interfacial processes, such as the capture of CO_2_ from the air^18^ or electron transfer in batteries,^19^ govern the performance of the RTIL-based devices.^8^ A central question concerning RTIL/water mixtures under salt-in-water and water-in-salt conditions is their microscopic interfacial structure, because this structure affects interfacial potentials,^8,20−22^ controls the double-layer capacitance,^23^ and determines the chemical reactivity. For neat RTILs, experimental techniques, including force–distance measurements^24^ and X-ray measurements,^25−27^ have shown that alternating positively and negatively charged layers form near interfaces. However, many questions regarding the microscopic interfacial structure of RTIL/water mixture interfaces remain elusive, because only a limited number of experimental approaches can provide molecular specificity; for RTIL/water mixtures, one should probe at least two species, water and ions, separately. In addition to molecular specificity, surface specificity is required to resolve the microscopic structure of these interfaces.

Heterodyne-detected sum-frequency generation (HD-SFG) spectroscopy is well-suited to provide information about the interfacial structure of RTIL ions and water separately via second-order nonlinear optical susceptibility [χ^(2)^]. In HD-SFG, a signal is generated by overlapping infrared and visible beams at the sample position, which then interferes with a local oscillator beam. The imaginary part of the complex χ^(2)^ [Imχ^(2)^] is non-zero at the interface and zero in centrosymmetric media, like bulk isotropic liquids. Thus, Imχ^(2)^ is interface-specific. Second, Imχ^(2)^ spectra can provide molecule-specific information, because the Imχ^(2)^ amplitude is enhanced when the infrared pulse frequency is resonant with vibrational modes of interfacial molecules. Third, the sign of Imχ^(2)^ peaks directly reflects the up-or-down orientation of these interfacial molecules relative to the plane of the interface. With these three advantages, HD-SFG can provide detailed insights into the interfacial structure of water and RTIL ions.

Conventional SFG, which detects |χ^(2)^|^2^ and, unlike HD-SFG, lacks phase information, has been used to probe the interfacial structure of RTILs.^11,28−37^ For example, Baldelli and co-workers probed the C–H stretch mode of the −CH_3_ group and discussed the possible conformation of [C_*n*_mim]^+^.^30−33^ Braunschweig, Smiatek, and co-workers combined simulations and experiments to clarify the interfacial conformation of RTIL/water mixtures.^34^ However, conventional SFG spectra could not provide information about the net orientation of the interfacial molecules. To fully unveil the interfacial structure^38,39^ of the air/RTIL/water mixture interface, heterodyne detection of SFG is essential.

In this study, we explore the molecular structure at the open surface of 1-alkyl-3-methylimidazolium tetrafluoroborate [C_*n*_mim][BF_4_]/water mixtures for *n* values of 2, 4, and 10, using HD-SFG spectroscopy. Our SFG data reveal the intricate behavior at the [C_*n*_mim][BF_4_]/water solution interface, as evidenced by various positive and negative peaks of the O–H stretch mode of water, depending on the mixture composition. We find that at low concentrations of RTIL (salt-in-water condition) [C_*n*_mim]^+^ appears at the topmost layer and the O → H group of interfacial water is oriented down toward the bulk solution. This water orientation is randomized with an increasing RTIL concentration due to the appearance of [BF_4_]^−^ at the topmost layer. At high concentrations of RTIL (water-in-salt conditions), the water molecules are isolated with their O–H group down-oriented. Furthermore, the comparison of different [C_*n*_mim]^+^ RTILs indicates that the length of the alkyl chain critically affects the ordering of the water molecules. Our results demonstrate that the interfacial water structure of ionic liquid/water mixtures can be tuned through the RTIL concentration.

First, to investigate the surface adsorption of [C_2_mim][BF_4_] and [C_4_mim][BF_4_], we performed surface tension measurements of the air/RTIL/water mixture interfaces. Figure 1 shows that the surface tension decreases significantly with an increase in the RTIL molar fractions (*x*_IL_), indicating surface adsorption of cations and anions. Although the surface tension of the RTIL/water mixture is drastically reduced by the addition of RTIL for mole fractions *x*_IL_ of <0.01 (*x*_IL_ ≤ 1.2 × 10^–3^ for [C_2_mim][BF_4_], and *x*_IL_ ≤ 1.0 × 10^–4^ for [C_4_mim][BF_4_]), the composition has a very limited impact on the surface tension of the water/RTIL mixtures at *x*_IL_ values of >0.01, consistent with the previous literature.^40,41^ This trend is similar to the concentration-dependent surface tension variation of surfactants in water.^42^ A question arising here is how the interfacial molecular structure of the water/RTIL mixture changes under the salt-in-water and water-in-salt conditions.

**Figure 1:**
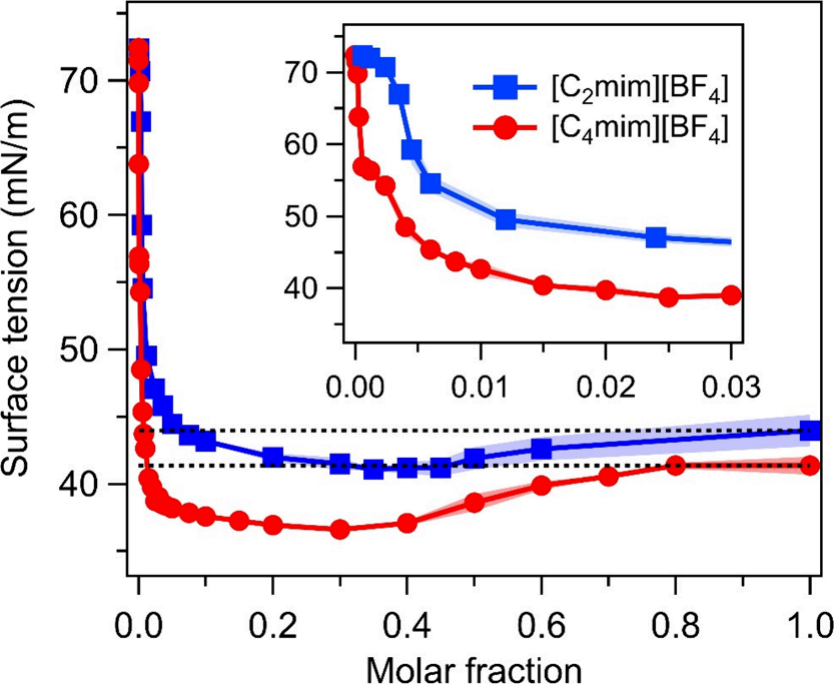
Surface tension data of the [C_2_mim][BF_4_]/water and [C_4_mim][BF_4_]/water mixture solutions vs the mole fraction of RTIL (*x*_IL_). The shaded region represents the error bar.

To obtain the reference spectra of pure water and RTIL, we measured the Imχ^(2)^ spectra of the air/neat water and air/neat [C_*n*_mim][BF_4_] interfaces. The resulting Imχ^(2)^ spectra for these samples are shown in Figure 2a. First, we focus on the Imχ^(2)^ spectrum at the air/water interface. The spectrum shows a sharp positive peak at 3700 cm^–1^, representing the dangling O–H groups pointing up, sticking out of the water interface.^43^ It further shows a broad negative band below 3550 cm^–1^, which originates from the hydrogen-bonded O–H groups of the interfacial water pointing down to the bulk.^44^ We found no spectral signatures below 3150 cm^–1^.

**Figure 2:**
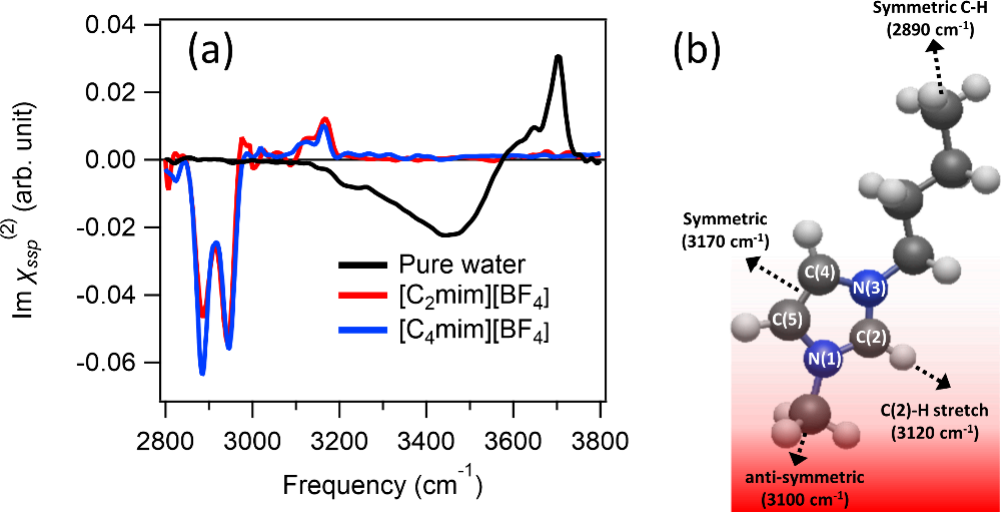
(a) Imχ^(2)^ spectra at the air interface of pure water, pure [C_2_mim][BF_4_], and pure [C_4_mim][BF_4_]. (b) Schematic figure of the structure of interfacial [C_4_mim]^+^ at the air/[C_4_mim][BF_4_] interface. The black arrows indicate the dipole directions of the symmetric and antisymmetric C–H stretch modes, respectively.

In contrast to the air/water interface, the air/[C_2_mim][BF_4_] and air/[C_4_mim][BF_4_] interfaces exhibit spectral features only below 3200 cm^–1^ originating from the various C–H stretch modes. The negative 2890 and 2940 cm^–1^ peaks arise from the symmetric C–H stretch mode and the Fermi resonance of the C–H stretch and H–C–H bending modes of the (C)–CH_3_ group, respectively.^11,45^ The negative signs of these peaks indicate that the ethyl (butyl) group of [C_2_mim]^+^ ([C_4_mim]^+^) points up toward the air.^45^ A positive 2980 cm^–1^ signature can be attributed to the antisymmetric C–H stretch mode of the (C)–CH_3_ group,^34,46−49^ while a negative dip at 3100 cm^–1^ is attributed to the antisymmetric C–H stretch mode of the (N)–CH_3_ group (Figure 2b).^11,32^ Note that the negative dip at 3100 cm^–1^ is smaller in the Imχ^(2)^ spectra of the [C_4_mim][BF_4_]/water mixture than in the Imχ^(2)^ spectra of the [C_2_mim][BF_4_]/water mixture, suggesting that the antisymmetric C–H stretch mode of the (N)–CH_3_ group is more parallel to the surface in [C_4_mim][BF_4_] than in [C_2_mim][BF_4_]. In addition, we observe positive 3120 and 3170 cm^–1^ SFG features. The 3120 and 3170 cm^–1^ features are assigned to the C(2)–H stretch and symmetric C–H stretch modes of the H–C(4)–C(5)–H group, respectively.^11,29,50−52^ Our ab initio calculation suggested that the positive symmetric C–H stretch mode of the H–C(4)–C(5)–H group and the positive C(2)–H stretch mode reflect the C(2) → H group pointing up to the air (see the Supporting Information). The appearance of these SFG features is consistent with the previous homodyne-detected SFG data,^31,32,34,46,53^ indicating that the sample quality is ensured. The structure of interfacial [C_4_mim]^+^ inferred from these signals is displayed in Figure 2b.

Subsequently, we measured the Imχ^(2)^ spectra at the air/[C_2_mim][BF_4_]/water mixture interfaces by varying the RTIL molar fractions (*x*_IL_). The data are shown in Figure 3a–e. Figure 3a shows the Imχ^(2)^ spectrum at an *x*_IL_ of 1.2 × 10^–3^. The addition of small amounts of [C_2_mim][BF_4_] drastically enhances the negative 3050–3500 cm^–1^ O–H stretch band and results in the appearance of a positive O–H stretch band at 3600 cm^–1^, the negative antisymmetric C–H band of the (N)–CH_3_ group at 3100 cm^–1^, and the C–H stretch bands of the −CH_3_ group at 2800–3000 cm^–1^. Furthermore, the free O–H peak disappears upon addition of [C_2_mim][BF_4_] to water. The negative sign of the 3050–3500 cm^–1^ O–H band indicates that these interfacial water molecules point down to the bulk solution. This down orientation can be explained from [C_2_mim]^+^ located at the topmost layer evidenced by the C–H stretch peak (Figure 3f). The sign of the positive O–H stretch band at 3600 cm^–1^ is the opposite of that of the 3050–3500 cm^–1^ O–H stretch band, which can be assigned to a OH stretch mode of water molecules weakly hydrogen-bonded to [BF_4_]^−^.^54−56^ The positive sign indicates that water molecules are located below the anion and point up to the bulk (Figure 3f). In fact, the Imχ^(2)^ spectrum an an *x*_IL_ of 1.2 × 10^–3^ resembles that of the positively charged lipid DPTAP at aqueous interfaces, for which, similarly, positively charged lipid head groups accumulate at the interface.^57^

**Figure 3:**
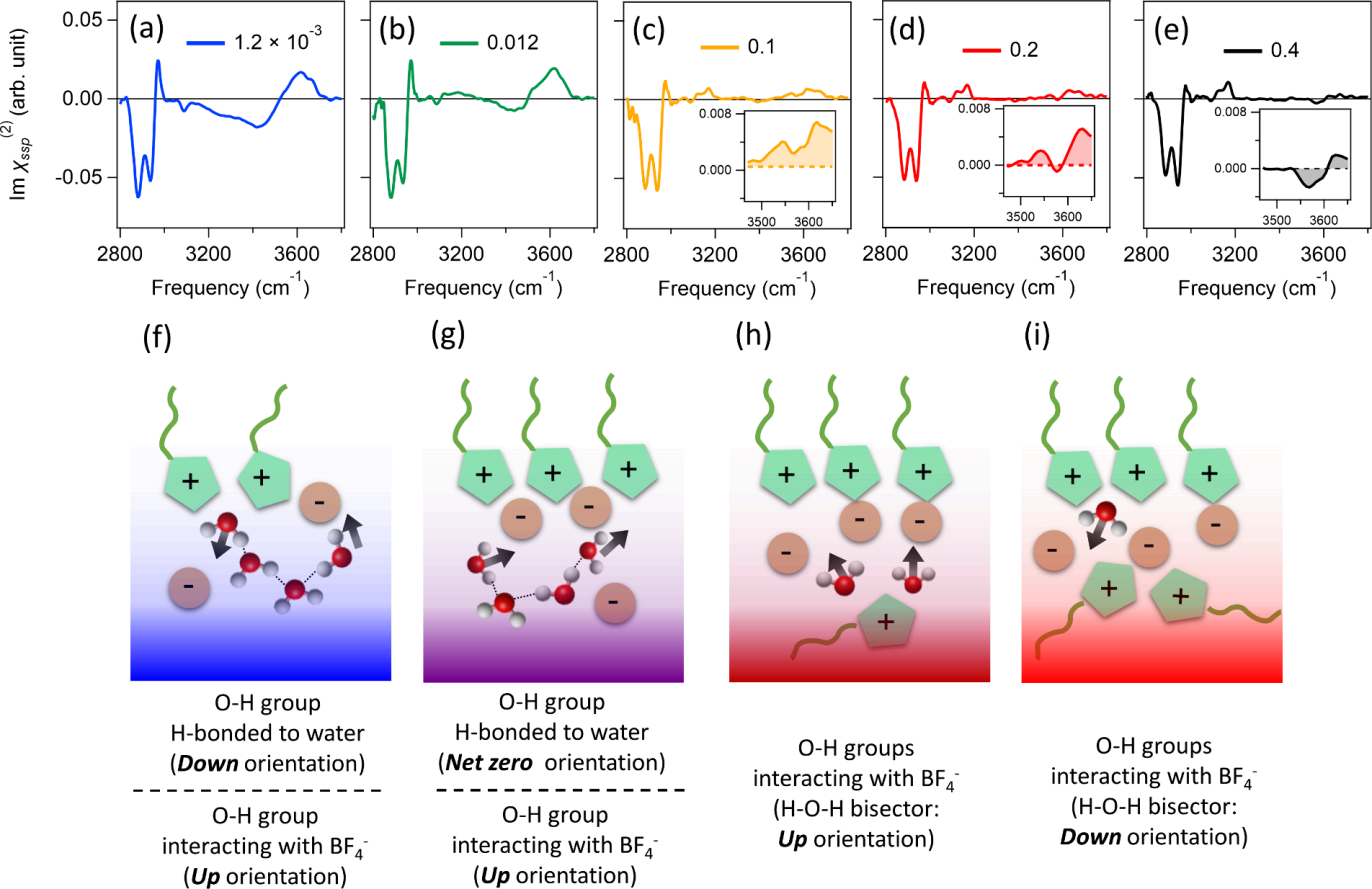
Variation of the Imχ^(2)^ spectra at the air/[C_2_mim][BF_4_]/water mixture solution interface for *x*_IL_ values of (a) 1.2 × 10^–3^, (b) 0.012, (c) 0.1, (d) 0.2, and (e) 0.4. The insets zoom in on the signals in the high-frequency region (3470–3660 cm^–1^) of the O–H stretch region. (f–i) Schematics of the interfacial structure of the [C_2_mim][BF_4_]/water mixture solution. The black arrows indicate the dipole directions of the interfacial water molecules. In panel f, the water molecule pointing up provides a 3580 cm^–1^ positive peak in panel a while the water molecule pointing down provides a negative band.

Figure 3b shows the Imχ^(2)^ spectrum at an *x*_IL_ of 0.012. With an increase in *x*_IL_ from 1.2 × 10^–3^ to 0.012, part of the negative O–H stretch band becomes positive, resulting in positive 3200 and 3600 cm^–1^ peaks together with a dip at 3450 cm^–1^. The Imχ^(2)^ spectrum at an *x*_IL_ of 0.012 resembles the spectrum of the aqueous interface in the presence of a mixture of positively charged DPTAP and negatively charged DPPG lipids (see Figure S1),^45^ indicating that both [C_4_mim]^+^ and [BF_4_]^−^ cover the water surface and the surface is less positively charged. Cations and anions start to form the interfacial molecular layering structures (Figure 3g), as is inferred by the molecular dynamics simulation and X-ray studies.^58−60^ Covering the water surface with an RTIL is consistent with a decrease in surface tension. Figure 3c shows the Imχ^(2)^ spectrum at an *x*_IL_ of 0.1. No negative peak in the frequency region of 3000–3500 cm^–1^ is discernible, but we observe a positive 3580 cm^–1^ peak and a positive 3620 cm^–1^ peak. These two peaks can be assigned to the symmetric and antisymmetric stretch modes, respectively, of H_2_O molecules.^54,61−63^ Both of the O–H groups are hydrogen-bonded to [BF_4_]^−^ but do not interact with other water molecules.^6,11,34^ Note that these symmetric and antisymmetric O–H stretches are different from the O–H stretch of the positive 3600 cm^–1^ peak seen in Figure 3a; in the diluted RTIL cases, a majority of the water molecules have water molecules as neighbors, and therefore, it is unlikely that both O–H groups would interact with [BF_4_]^−^ (see Figure S9).

The presence of these very weak SFG signatures of water in the water-in-salt solution was pointed out in a previous |χ^(2)^|^2^ study.^34^ However, these |χ^(2)^|^2^ data could not resolve the symmetric/antisymmetric splitting, whereas our HD-SFG spectra clearly show the spectral characteristics of confined water. The observation of the symmetric and antisymmetric vibrations indicates that the interfacial water molecules are confined by the RTIL ions. The sign of the peaks reflects the direction of the transition dipole moment. For the symmetric O–H stretch, the direction of the transition dipole moment parallels the bisector of the H–O–H angle, while for the antisymmetric O–H stretch, it points from one hydrogen atom (H_1_) to the other hydrogen atom (H_2_). The positive symmetric O–H peak at 3580 cm^–1^ indicates that the O–H groups of the interfacial water pointing up to the air (Figure 3h) are indicative of these molecules interacting with [BF_4_]^−^ at the interface. With a further increase in the RTIL concentration from 0.2 to 0.4 (Figure 3d,e) toward the water-in-salt condition, the sign of this 3580 cm^–1^ peak changes. The negative symmetrical O–H peak indicates that such confined water molecules point down to the bulk at increased RTIL concentrations. As such, the interfacial water molecules flip their orientation upon variation of the RTIL concentration (Figure 3i). Note that the sign of the antisymmetric O–H peak at 3620 cm^–1^ remains the same, but the amplitude decreases, indicating that the direction of the H_1_ → H_2_ vector does not flip but becomes more parallel to the surface.

The SFG data of the [C_4_mim][BF_4_]/water mixtures are shown in Figure 4a–d. The data with an *x*_IL_ of 3 × 10^–5^ in Figure 4a show a negative 3050–3500 cm^–1^ band and a positive 3620 cm^–1^ peak. With an increase in *x*_IL_ from 3 × 10^–5^ to 0.0012, we find that the negative hydrogen-bonded O–H band decreases and the positive 3200 cm^–1^ peak appears, similar to our findings for [C_2_mim][BF_4_]/water mixtures (Figure 4b). At an *x*_IL_ of 0.2, the spectral features of the O–H groups at 3450–3700 cm^–1^ disappear (see Figure S5), indicating that the water molecules are excluded from the surface. With an increase in *x*_IL_ from 0.2 to 0.4, the spectral features remain similar. In contrast to the case for the [C_2_mim][BF_4_]/water mixtures discussed above, peaks in the frequency region of 3500–3650 cm^–1^ are, if present, similar in intensity to the noise of the spectra (see the insets of panels c and d of Figure 4 and Figure S5). Note that the symmetric C–H peak at 2890 cm^–1^ in the SFG spectra of the [C_2_mim][BF_4_]/water and [C_4_mim][BF_4_]/water mixtures (Figures 3a–e and 4a–d) is insensitive to *x*_IL_. The observation indicates that the number density and orientation of the alkyl chains of [C_2_mim]^+^ and [C_4_mim]^+^ remain unchanged at the topmost layer, once the [C_2_mim]^+^/[C_4_mim]^+^ monolayer is formed (Figure 3f–i).

**Figure 4:**
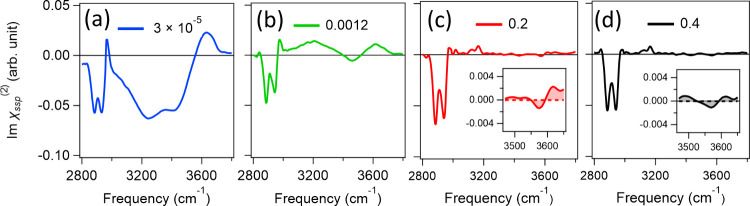
Variation of the Imχ^(2)^ spectra at the air/[C_4_mim][BF_4_]/water mixture solution interface for *x*_IL_ values of (a) 3 × 10^–5^, (b) 0.0012, (c) 0.2, and (d) 0.4.

For the air/[C_10_mim][BF_4_]/water mixture interface, again, spectral signatures reminiscent of a positively charged surfactant/water interface can be seen at an *x*_IL_ of 1 × 10^–5^, similar to the case for the [C_2_mim][BF_4_] and [C_4_mim][BF_4_] samples (Figure 5). A further increase in *x*_IL_ results in the macroscopic phase separation of [C_10_mim][BF_4_] and water (see Figure S8). These results indicate that cations with longer alkyl chains enhance the alignment of the interfacial water and promote macroscopic phase separation.

**Figure 5:**
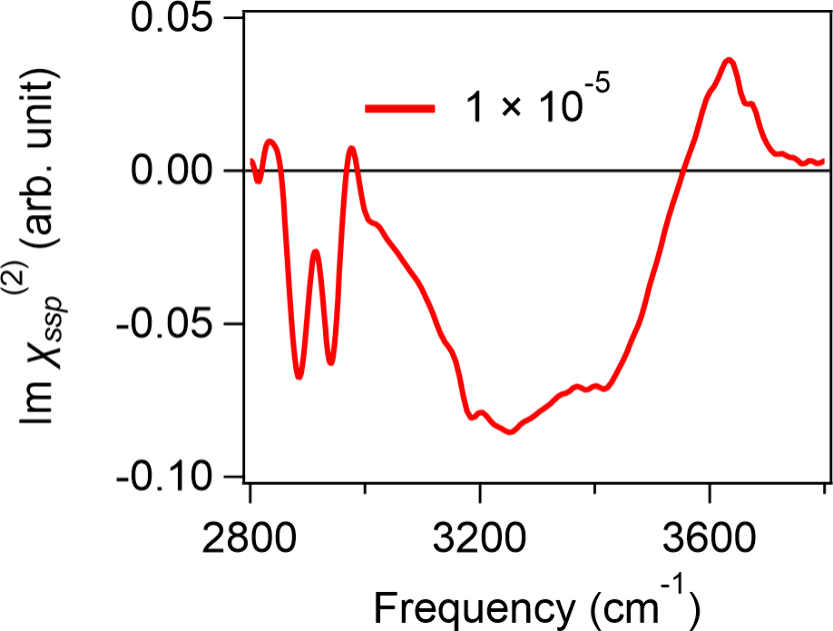
Imχ^(2)^ spectra at the air interface of the [C_10_mim][BF_4_]/water mixture at an *x*_IL_ of 1 × 10^–5^.

Now, we compare the Imχ^(2)^ spectra of different [C_*n*_mim][BF_4_] samples. First, we compared the data with the most pronounced negative 3050–3500 cm^–1^ O–H stretch band, for which the surface is occupied by [C_*n*_mim]^+^ and the density of [BF_4_]^−^ at the surface is minimized (Figure 3f) under the salt-in-water condition. The data are shown in Figure 6a. Although the C–H peak amplitudes of the alkyl chain are comparable for these mixtures, the magnitude of the negative O–H stretch peak increases with alkyl chain length *n* of [C_*n*_mim]^+^. Furthermore, the maximum in the negative band is reached at a lower *x*_IL_ for a higher *n* (see the Supporting Information). These observations suggest that the increased hydrophobicity of [C_*n*_mim]^+^ with an increase in *n* results in an increased surface propensity of [C_*n*_mim]^+^, allowing for higher coverage of [C_*n*_mim]^+^ at small *x*_IL_ values. When the bulk ion concentration, including the concentration of [BF_4_]^−^ counterions, is low, the Debye length is long, and the electric field created by the [C_*n*_mim]^+^ layer at the topmost solution surface is only weakly screened. Hence, the electric field breaks the centrosymmetry across a larger volume, and the diffuse double layer contributes to the Imχ^(2)^ spectra, as the so-called “χ^(3)^ contribution”.^64,65^

**Figure 6:**
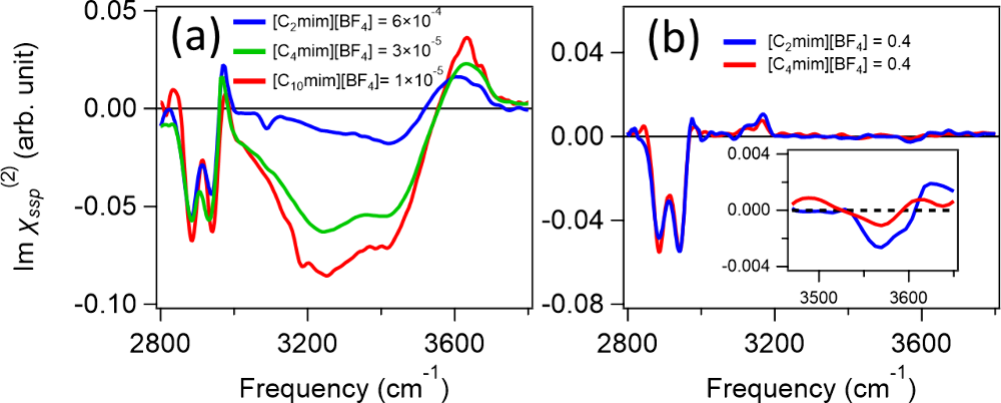
Comparison of the Imχ^(2)^ spectra at the air/[C_*n*_mim][BF_4_] interfaces with *n* values of 2, 4, and 10: (a) salt-in-water condition (*x*_IL_ < 6 × 10^–4^) and (b) water-in-salt condition (*x*_IL_ = 0.4).

Next, we focus on the water-in-salt condition. We compare the Imχ^(2)^ spectra of the [C_2_mim][BF_4_]/water mixture to that of the [C_4_mim][BF_4_]/water mixture at an *x*_IL_ of 0.4. The data are shown in Figure 6b. Although the molar concentrations of water for these samples are similar (8.3 and 7.0 M for [C_2_mim]^+^ and [C_4_mim]^+^, respectively) and the C–H stretch peaks in the range of 2800–3200 cm^–1^ are similar, the signal from the confined water differs substantially between these samples. Water signals are more prominent in the [C_2_mim]^+^ sample than in the [C_4_mim]^+^ sample. This indicates that water molecules tend to be excluded from the interfacial region with an increase in alkyl chain length, as reported by the recent molecular dynamics simulation.^34^ This exclusion of water molecules from the interface arises from the more crystalline structure of [C_*n*_mim][BF_4_] with longer alkyl chains (larger *n*); in fact, [C_*n*_mim][BF_4_] with longer alkyl chains (larger *n*) tends to have a more crystal-like interfacial structure with a marked oscillation of the charge density along the surface normal.^27^ Furthermore, this more crystal-like structure induces greater molecular packing and hence decreases the surface tension of pure RTIL.

In summary, using surface- and orientation-sensitive vibrational HD-SFG spectroscopy, we find that the interfacial water orientation flips with varying RTIL concentrations at the air/RTIL/water mixture interface. For the salt-in-water condition, the [C_*n*_mim]^+^ of the [C_*n*_mim][BF_4_]/water mixture behaves as a surfactant. The cations form a positively charged monolayer, and interfacial water molecules point their OH groups down to the bulk solution. With an increase in the RTIL concentration, [BF_4_]^−^ appears at the topmost layer of the solution and, thus, the water reorients to face up toward the air. When the RTIL concentration is further increased and reaches the water-in-salt condition, the interfacial water molecules are confined by the RTILs and point again down to the bulk. The extent of the water orientation can be efficiently tuned by changing the alkyl chain of [C_*n*_mim]^+^. Such knowledge of the interfacial water structure is pivotal to understanding the electrochemical behavior, such as the double-layer capacitance of the RTIL/water mixtures. It may open a path to optimizing the hydrogen/oxygen evolution reaction under the water-in-salt and salt-in-water conditions. Furthermore, extending HD-SFG measurements from the air/liquid interface to the electrified interface using a graphene electrode enables the study of water-in-salt electrolytes in batteries.^66−69^ The electrode/electrolyte interface in the water-in-salt solution provides unique structural features, such as ion layer accumulation and desolvation processes,^23^ which have potential applications in batteries.

## Supporting Information Available

Sample preparation, details of HD and surface tension measurements, HD-SFG data of the mixed lipid/water interfaces, HD-SFG data of the [C*_n_*mim][BF_4_]/water mixture solution, and details of the calculation of the transition dipole moment and transition polarizability (PDF)
