# Supplementary material for: Flipping Water Orientation at the Surface of Water-in-Salt and Salt-in-Water Solutions
Source: J Phys Chem Lett. 2024 Oct 3;15(40):10265–71. doi: 10.1021/acs.jpclett.4c01834 (PMC11472344; doi:10.1021/acs.jpclett.4c01834)
Supplement: Supplementary file 1 — jz4c01834_si_001.pdf [file jz4c01834_si_001.pdf]

Supporting Information

Flipping water orientation at the surface of water-in-salt and salt-in-water solutions

Chun-Chieh Yu,[1,§] Kuo-Yang Chiang,[1,§] Ali Dhinojwala,[2] Mischa Bonn,[1] Johannes Hunger,[1] and Yuki Nagata[1,*]

1. Max Planck Institute for Polymer Research, Ackermannweg 10, 55128 Mainz, Germany

2. Department of Polymer Science, The University of Akron, Akron, Ohio 44325-3909, United States

Sample preparation

Ethyl-3-methylimidazolium tetrafluoroborate (>98%), 1-Butyl-3-methylimidazolium tetrafluoroborate (99%), and 1-Decyl-3-methylimidazolium tetrafluoroborate (>98%) were purchased from IoLiTec and used without further purification. The ultra-pure H_2O was obtained from a Milli-Q machine (18 MΩ-cm resistivity). The ionic liquid-water solutions were poured into a glass petri-dish with 5 cm diameter for the SFG measurement.

DPPG (sodium salt) and DPTAP (chloride salt) were purchased from Avanti Polar. Lipids were dissolved in a mixture of 90% chloroform (Fischer Scientific, stabilized with amylene, >99%) and 10% methanol (VWR Chemicals, 99.8%) at a concentration of 4.3×10^{-4} mol/L. The ultra-pure H_2O were poured into a Teflon trough. For generating the water/charged lipid interfaces, a controlled amount of lipid solution was added onto the water using a click syringe. The surface area per lipid was 39 $Å^2$.

Surface tension measurement

The surface tension is measured by a commercial tensiometer (KBN 315 Sensor Head, Kibron Inc.) based on the Wilhelmy method and utilizing a small-diameter alloy probe.

Heterodyne-detected SFG measurement

We used a collinear beam geometry using a Ti:Sapphire regenerative amplifier (Spitfire Ace, Spectra-Physics, centered at 800 nm, ~40 fs pulse duration, 5 mJ pulse energy, 1 kHz repetition rate).[1] A part of the output was used to generate a broadband infrared (IR) pulse in an optical parametric amplifier (Light Conversion TOPAS-C) with a silver gallium disulfide ($AgGaS_2$) crystal. The other part of the output was directed through a pulse shaper consisting of a grating-cylindrical mirror system to generate a narrowband visible pulse with a bandwidth of ~10 cm^{-1}. The IR and visible beams were firstly focused onto a 20 μm-thick *y*-cut quartz plate to generate a local oscillator (LO) signal. Then, these beams were collinearly passed through a 5 mm-thick $SrTiO_3$ plate for the phase modulation and were focused onto the sample surface at angles of incidence of 45° with pulse energies of ~6 μJ and ~3 μJ for visible and IR pulses, respectively. The SFG signal from the sample interfered with the SFG signal from the LO, generating the

SFG interferogram. The SFG interferogram was dispersed in a spectrometer (Teledyne Princeton Instruments, HRS-300) and detected by a liquid-nitrogen cooled CCD camera (Teledyne Princeton Instruments, PyLoN). During the measurements, the sample height was corrected based on a height displacement sensor (GL-82, Keyence).

The complex-valued second-order nonlinear susceptibility ($\chi^{(2)}$) from the samples were obtained via the Fourier analysis of the interferogram and normalization by that from a z-cut quartz crystal.[2,3] The measurements were performed with *ssp* (denoting *s*-, *s*-, and *p*-polarized SFG, visible, and IR beams, respectively) polarization combination. To avoid the effect of water vapor absorption of IR, the optical path of SFG setup was purged with dry air.

HD-SFG spectra of the mixed lipid/water interfaces

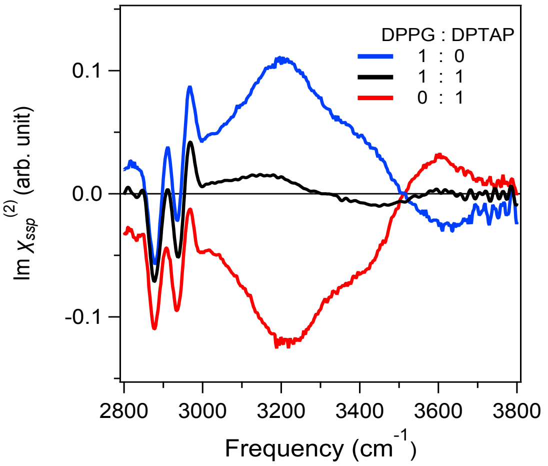

Figure S1. Im$\chi^{(2)}$ spectra of mixed lipid/water (H_2O) interfaces with different molar ratios of the DPPG and DPTAP lipids.

HD-SFG spectra of the [C$_n$mim][BF_4]-water mixture solution

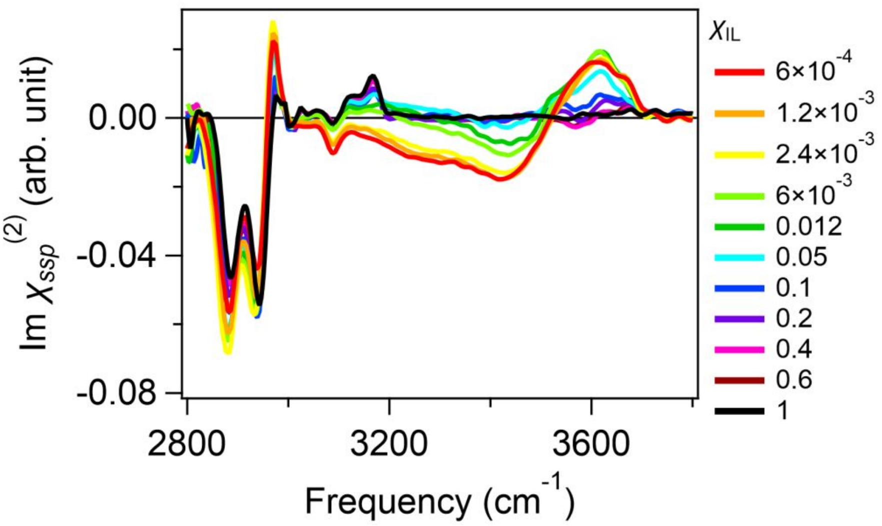

Figure S2. Variation of the Im$\chi^{(2)}$ spectra at the air/[C$_2$mim][BF_4]-water mixture solution interface.

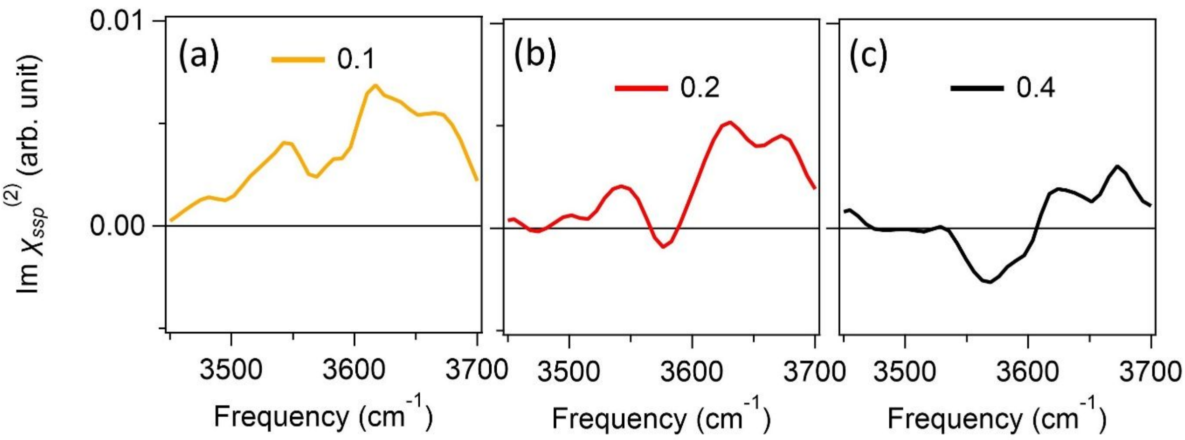

Figure S3. Im$\chi^{(2)}$ spectra for [C$_2$mim][BF_4]-water mixtures for $x_{IL} =$ (a) 0.1, (b) 0.2, and (c) 0.4, in the region of 3450 to 3700 cm^{-1}.

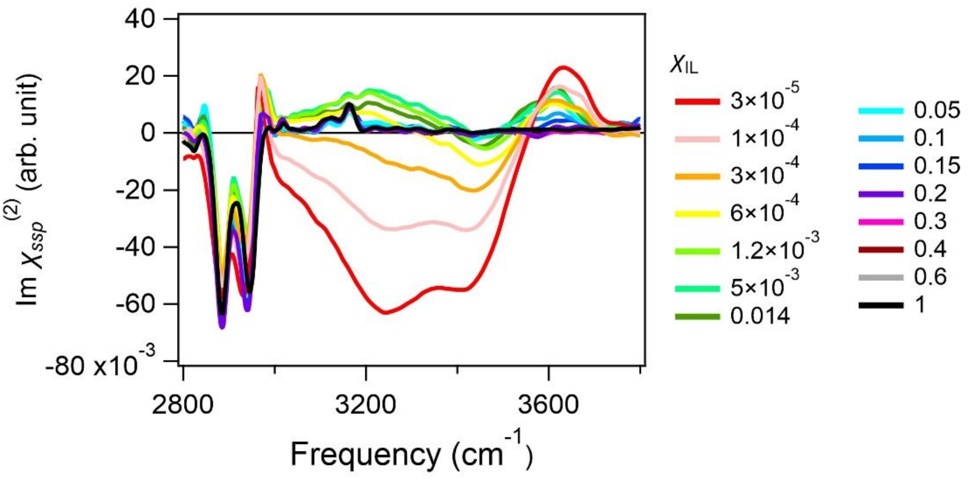

Figure S4. Variation of the Im$\chi^{(2)}$ spectra at the air/[C$_4$mim][BF$_4$]-water mixture solution interface.

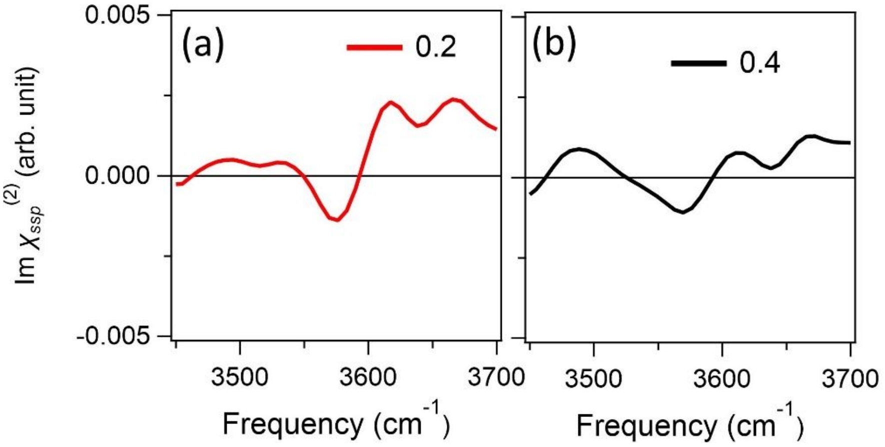

Figure S5. Im$\chi^{(2)}$ spectra for [C$_4$mim][BF$_4$]-water mixtures, for $x_{IL} =$ (a) 0.2 and (b) 0.4 in the region of 3450 to 3700 cm^{-1}.

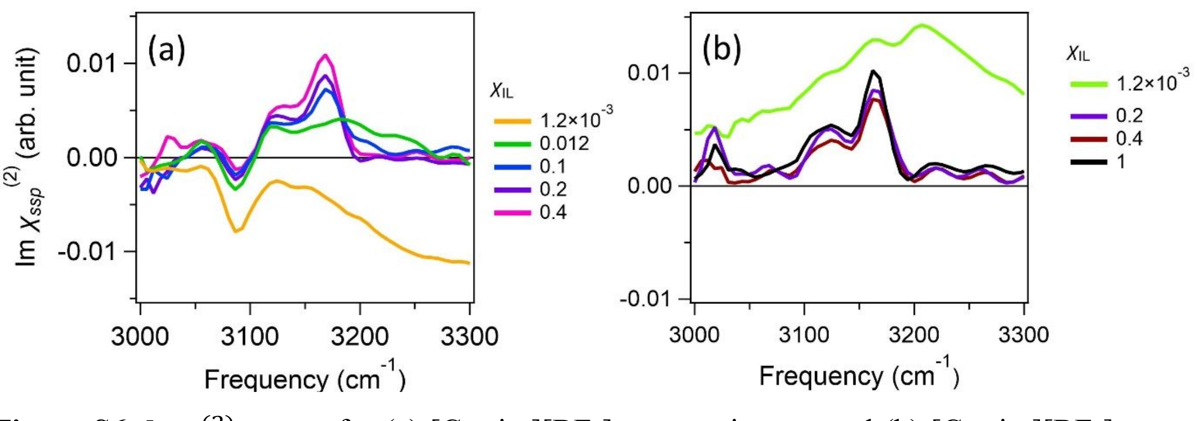

Figure S6. Im$\chi^{(2)}$spectra for (a) [C$_2$mim][BF$_4$]-water mixtures and (b) [C$_4$mim][BF$_4$]-water mixtures, in the region of 3000 to 3300 cm^{-1}.

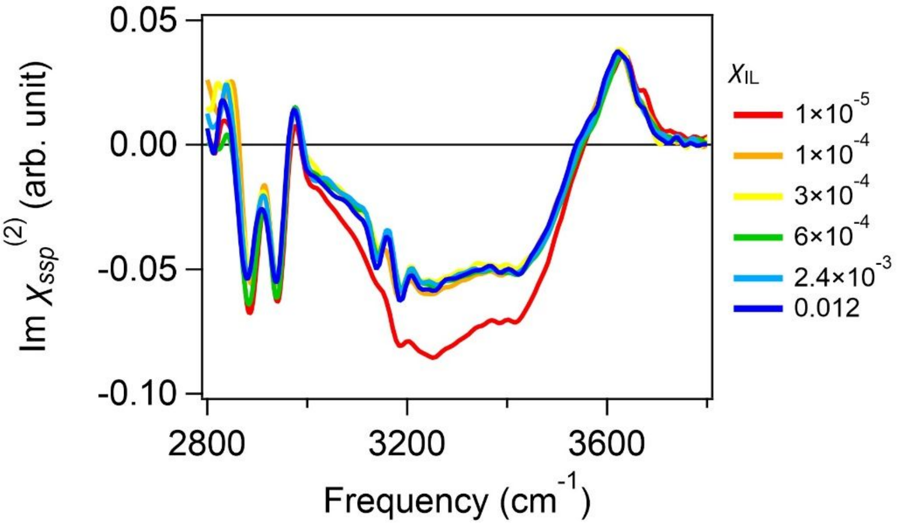

Figure S7. Variation of the Im$\chi^{(2)}$ spectra at the air/[C$_{10}$mim][BF$_4$]-water mixture solution interface.

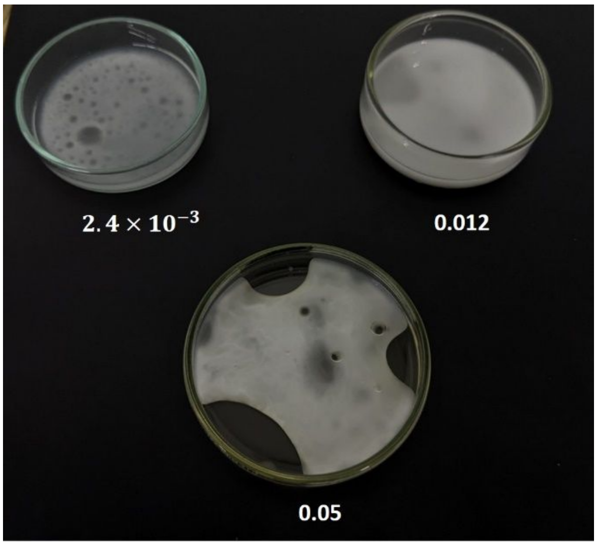

Figure S8. Macroscopic phase separation of [C$_{10}$mim][BF$_4$] and water at $x_{IL} = 2.4 \times 10^{-3}$, 0.012, and 0.05.

Transition dipole moment and transition polarizability

To connect the sign of the Im$\chi^{(2)}$ peaks of the imidazolium C-H symmetric and anti-symmetric modes with the molecular orientations, we explored the transition dipole moment and transition polarizability by carrying out *ab initio* calculations of $[C_2mim]^+$. The calculation was done at the CAM-B3LYP/aug-cc-pVTZ level of theory by using the ORCA quantum chemistry code.[4] The normal modes were computed by calculating the Hessian matrix and then obtained the transition dipole moment ($\Delta\mu$) and the transition polarizability ($\Delta\alpha$) of $[C_2mim]^+$ by displacing the atom positions along the normal modes.

The computed $\Delta\mu\Delta\alpha/\Delta E$ for the C(2)-H stretch mode which is proportional to the Im$\chi^{(2)}$ peak area for the corresponding vibrational mode is positive (negative), when C(2)→H points *down* to the bulk solution (*up* to the air). Here, ΔE is the energy difference of the molecule due to the displacement of the atoms. Furthermore, $\Delta\mu\Delta\alpha/\Delta E$ for the symmetric C-H stretch mode of the H-C(4)-C(5)-H group is also positive (negative), when C(2)→H points *down* to the bulk solution (*up* to the air). Since both peaks are positive for pure $[C_2mim][BF_4]$ and $[C_4mim][BF_4]$, we concluded that C(2)→H points *down* to the bulk solution.

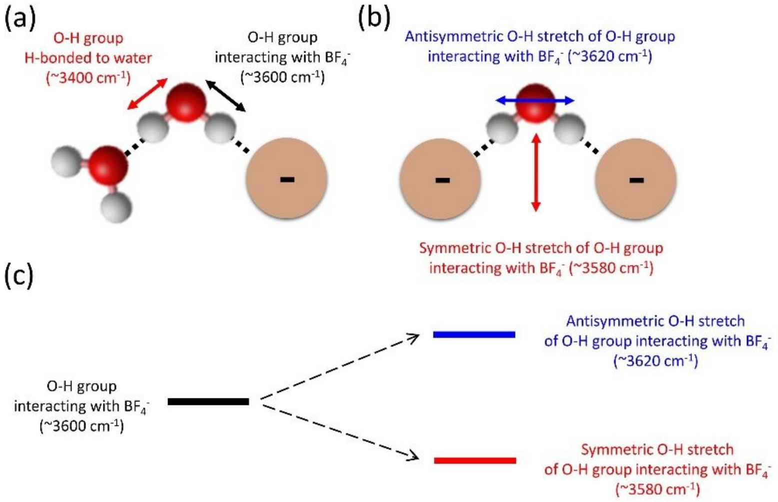

Figure S9. (a) The direction of the transition dipole moments for O-H groups hydrogen-bonded to water and interacting with the $[BF_4]^-$. (b) The direction of the transition dipole moments for symmetric (antisymmetric) O-H group interacting with the $[BF_4]^-$. (c) Energy level diagram of a single O-H group interacting with $[BF_4]^-$, and the symmetric (antisymmetric) O-H stretch group interacting with $[BF_4]^-$.

References

(1) Chiang, K. Y.; Seki, T.; Yu, C. C.; Ohto, T.; Hunger, J.; Bonn, M.; Nagata, Y. The Dielectric Function Profile across the Water Interface through Surface-Specific Vibrational Spectroscopy and Simulations. *Proc. Natl. Acad. Sci. U. S. A.* **2022**, *119* (36), e2204156119. https://doi.org/10.1073/pnas.2204156119.

(2) Nihonyanagi, S.; Mondal, J. A.; Yamaguchi, S.; Tahara, T. Structure and Dynamics of Interfacial Water Studied by Heterodyne-Detected Vibrational Sum-Frequency Generation. *Annu. Rev. Phys. Chem.* **2013**, *64* (1), 579–603. https://doi.org/10.1146/annurev-physchem-040412-110138.

(3) Shen, Y. R. Phase-Sensitive Sum-Frequency Spectroscopy. *Annu. Rev. Phys. Chem.* **2013**, *64* (1), 129–150. https://doi.org/10.1146/annurev-physchem-040412-110110.

(4) Neese, F. The ORCA Program System. *Wiley Interdiscip. Rev. Comput. Mol. Sci.* **2012**, *2* (1), 73–78. https://doi.org/10.1002/wcms.81.
